# Characterization of *Klebsiella pneumoniae* carrying the *bla*_NDM-1_ gene in IncX3 plasmids and the rare In1765 in an IncFIB-IncHI1B plasmid

**DOI:** 10.3389/fcimb.2023.1324846

**Published:** 2024-01-11

**Authors:** Liman Ma, Ying Qu, Wenji Wang, Dongguo Wang

**Affiliations:** ^1^School of Medicine, Taizhou University, Taizhou, Zhejiang, China; ^2^Department of Central Laboratory, Taizhou Municipal Hospital affiliated with Taizhou University, Taizhou, Zhejiang, China; ^3^Department of Clinical Medicine Laboratory, Taizhou Municipal Hospital Affiliated with Taizhou University, Taizhou, Zhejiang, China; ^4^School of Life Sciences, Taizhou University, Taizhou, Zhejiang, China

**Keywords:** *Klebsiella pneumoniae*, plasmid pA_F11, plasmid pB_F11, plasmid pC_F11, drug resistance, In1765, the composite structure of *ble*_MBL_ and *bla*_NDM-1_

## Abstract

**Background:**

Today, the *bla*_NDM_ gene is widely distributed on several plasmids from a variety of Gram-negative bacteria, primarily in transposons and gene cassettes within their multidrug-resistant (MDR) regions. This has led to the global dissemination of the *bla*_NDM_ gene.

**Methods:**

The determination of class A beta-lactamase, class B and D carbapenemases was performed according to the recommendations of the Clinical and Laboratory Standards Institute (CLSI). Antimicrobial susceptibility testing was performed using both the BioMerieux VITEK2 system and antibiotic paper diffusion methods. Plasmid transfer was then evaluated by conjugation experiments and plasmid electroporation assays. To comprehensively analyze the complete genome of *K. pneumoniae* strain F11 and to investigate the presence of mobile genetic elements associated with antibiotic resistance and virulence genes, Nanopore and Illumina sequencing platforms were used, and bioinformatics methods were applied to analyze the obtained data.

**Results:**

Our findings revealed that *K. pneumoniae* strain F11 carried class A beta-lactamase and classes B+D carbapenemases, and exhibited resistance to commonly used antibiotics, particularly tigecycline and ceftazidime/avibactam, due to the presence of relevant resistance genes. Plasmid transfer assays demonstrated successful recovery of plasmids pA_F11 and pB_F11, with average conjugation frequencies of 2.91×10^-4^ and 1.56×10^-4^, respectively. However, plasmids pC_F11 and pD_F11 failed in both conjugation and electroporation experiments. The MDR region of plasmid pA_F11 contained rare In1765, Tn*As2*, and Tn*As3* elements. The MDR2 region of plasmid pB_F11 functioned as a mobile genetic “island” and lacked the *bla*_NDM-1_ gene, serving as a “bridge” connecting the early composite structure of *ble*_MBL_ and *bla*_NDM-1_ to the recent composite structure. Additionally, the MDR1 region of plasmid pB_F11 comprised In27, Tn*As1*, Tn*As3*, and Tn*2*; and plasmid pC_F11 harbored the recent composite structure of *ble*_MBL_ and *bla*_NDM-1_ within Tn*3000* which partially contained partial Tn*125*.

**Conclusion:**

This study demonstrated that complex combinations of transposons and integron overlaps, along with the synergistic effects of different drug resistance and virulence genes, led to a lack of effective therapeutic agents for strain F11, therefore its dissemination and prevalence should be strictly controlled.

## Introduction

NDM-producing bacteria exhibited the ability to hydrolyze a wide range of beta- lactam antibiotics, except for monolactams (Nordmann et al., 2011), consequently, therapeutic options for infections caused by NDM-producing strains were severely limited, resulting in significantly higher mortality rates among infected patients (Logan and Weinstein, 2017; Guducuoglu et al., 2018). The *bla*_NDM-1_ gene was commonly associated with composite genes that included the aminoglycoside resistance gene *aphA6* and the metallo-beta-lactamase (MBL) gene. Typically, these composite genes were located adjacent to either intact or truncated copies of IS*Aba125* (Toleman et al., 2012). The *bla*_NDM-1_ gene was almost always found in association with mobile genetic elements (Loh et al., 2020), contributing to its evolutionary potential and rapid dissemination (Wu et al., 2019). As a result, the *bla*_NDM-1_ gene had emerged as a major global public health concern (Walsh et al., 2011; Dortet et al., 2014). Among NDM-positive *Enterobacteriaceae* bacteria, *K. pneumoniae* accounted for over half of the cases, followed by *Escherichia coli* and the *Enterobacter cloacae* complex (Wu et al., 2019).

Furthermore, the presence of NDM had also been detected in other species, including *Acinetobacter* and *Pseudomonas* (Costa et al., 2021). Poirel et al. proposed a hypothesis suggesting that the initial integration of the *bla*_NDM_ gene occurred within the chromosome of *Acinetobacter* originated from an unidentified environmental species. Subsequently, two copies of the insertion sequence (IS) IS*Aba125* captured the *bla*_NDM_ gene, leading to the formation of the IS*Aba125* complex transposon known as Tn*125* (Poirel et al., 2012). Through the transfer of the Tn*125* transposon, the *bla*_NDM_ gene disseminated among *Enterobacteriaceae*, *Acinetobacter*, and *Pseudomonas*. Consequently, both the *bla*_NDM_ and its Tn*125* components could be found within MDR regions of relevant bacterial plasmids or chromosomes, exhibiting various truncations and deletions (Wu et al., 2019). Notably, the truncated insertion sequence IS*Aba125* consistently appeared upstream of *bla*_NDM_, while the bleomycin resistance gene (*ble*_MBL_) emerged downstream of it. These genetic features were commonly observed in other typical contexts where *bla*_NDM_ was present (Wu et al., 2019). Additionally, the expression of *ble*_MBL_ and *bla*_NDM_ was driven by the same promoter, resulting in their co-expression (Dortet et al., 2017).

In addition to transposons, the involvement of other mobile genetic elements, including IS elements and integrons, had been implicated in the mobilization of NDM (Guducuoglu et al., 2018). The rapid dissemination of these mobile genetic elements had contributed to the unprecedented and widespread prevalence of NDM, posing a significant threat to global healthcare. Consequently, there was an urgent need to focus on controlling the potentially dangerous proliferation of NDM within hospital settings. To date, a total of 31 subtypes of NDM had been identified, with NDM-1 remaining the most prevalent worldwide (https://www.bldb.eu). NDM-1 was widely distributed among various *Enterobacteriaceae* and other Gram-negative bacteria, while NDM-5 appeared to be more prevalent in *E. coli* (Wu et al., 2019). The ability of NDM-5 to efficiently dissemination among bacteria carrying incompatible plasmids was a significant worries (Bonnin et al., 2012), raising alarm about the dissemination of high-risk clones (Costa et al., 2021). Moreover, NDM-5 exhibited higher levels of antibiotic resistance compared to NDM-1 (Hornsey et al., 2011), posing a particular challenge for clinical treatment (Costa et al., 2021). NDM-producing strains had disseminated the gene to a wide range of Gram-negative bacteria via MDR plasmids, leading to serious concerns that patients infected with these bacteria might be left without effective treatment options.

In this study, the main focus was to compare and analyze the F11_plasmids, which carried the NDM-1 gene along with the rare integron In1765, in comparison with related plasmids. The study aimed to characterize the structure of these plasmids and to investigate the evolutionary processes associated with NDM.

## Materials and methods

### Bacterial strains and sequencing of the 16S rRNA gene

*K. pneumoniae* F11 strain was isolated from the sputum of an hospitalized patient in our hospital in 2020. EC600 and *E. coli* DH5α were used as hosts for cloning. The almost complete 16S rRNA gene for *K. pneumoniae* F11 strain was amplified by PCR using the following primers AGAGTTTGATYMTGGCTCAG (forward) and TACCTTGTTACGACTT (Y, T, or C; M, A, or C) (reverse). The PCR reaction included a Taq enzyme, which was a mixture of Fermentas Taq and Pfu enzymes in a ratio of 3:1 (ThermoFisher Scientific, Burlington, VT, USA). Each 30 ml reaction contained 1.5 U of the enzyme. The PCR amplification was carried out using a temperature program consisting of 30 cycles. The program started with an initial denaturation step at 94°C for 3 minutes, followed by denaturation at 94°C for 40 seconds, annealing at 50°C for 40 seconds, extension at 72°C for 1 minute, and a final extension step at 72°C for 5 minutes. The length of the amplified fragment was approximately 1,500 bp (Frank et al., 2008). PCR products were confirmed by sequencing in both directions.

### Experiments of conjugal transfer and plasmid transfer

#### Conjugation experiments

In accordance with a previously reported method (Chen et al., 2015), conjugation experiments were conducted using lysogeny broth (LB) as the medium. The recipient strain was strain EC600 (TaKaRa, China), while the donor was strain F11. In LB cultures, both donor and recipient bacteria experienced a brief lag phase of 0.5 to 1 hour before growth begins. Between 1.5 and 4 hours, the cultures were in the logarithmic phase and then enter the stationary phase at about 4 hours. To initiate the conjugation process, 0.5 mL of logarithmic phase cultures of both donor and recipient cells were mixed together in a total volume of 4 mL of fresh LB medium. The mixture was then incubated at a temperature of 35°C for a duration of 18 to 24 hours without shaking. Following the incubation period, transconjugants were selected by plating the conjugation mixture onto trypticase soy agar (TSA) plates supplemented using 1 ml with 10 μg/L of rifampicin and 0.02 μg/L of imipenem. These antibiotics were included in the selection medium to inhibit the growth of the donor and recipient strains, while allowing the growth of the transconjugants that acquired the resistance genes during conjugation.

#### Plasmid-electroporation assays

In the transformation experiments, *E. coli* DH5α cells (TaKaRa, China) were used as the recipient cells for plasmid electroporation. The conjugation frequency was calculated as the number of transconjugants obtained per initial donor cell. To prepare electrocompetent cells for electroporation, the bacteria were grown until they reaching an optical density at 600 nm (OD_600_) of 0.5–0.6. Cells were then harvested by centrifugation at 4°C. Two rounds of washing and centrifugation were performed at 4°C using 1 volume of milliQ water, followed by centrifugation at 6,000 rpm (4,025×g). In the final wash step, the cells were resuspended in 1/50 volume of 10% glycerol. The cells were then aliquoted into 50 μL samples, frozen on dry ice, and stored at -70°C until further use.

For the electroporation step, aliquots of the electrocompetent cells were thawed and mixed with less than 10 ng of DNA in a 0.2 cm cuvette (Bio-Rad, California, USA). The cell-DNA mixture was subjected to an electrical pulse using specific parameters: 2.5 kV, 25 mF, and 200 Ω in a MicroPulser (Bio-Rad, California, USA). Following electroporation, the cells were added to 1 mL of LB medium and incubated at 37°C with shaking (225 rpm, 5.660×g) for 1 hour to allow for antibiotic expression. After the incubation period, the transformed cells were plated onto selective media containing antibiotics. When strain F11 plasmids were used in electroporation or transconjugation experiments, appropriate selection was performed using 1 ml with 10 μg/L of rifampicin and 0.02 μg/L of imipenem.

#### Detection of Class A beta-lactamase, Classes B carbapenemase and Class D carbapenemase

The detection of class A beta-lactamase, class B carbapenemase, and class D carbapenemase was performed using different methods recommended by CLSI (CLSI, 2022). These methods involved the use of paper discs containing specific inhibitors or substrates, which were placed on an agar plate inoculated with the test strain. The growth of the strain around the discs indicated the presence of the corresponding enzyme.

To specifically detect class B carbapenemases (metallo-bete-lactamases), the modified carbapenem inactivation method (mCIM) and modified carbapenem inactivation+ EDTA (eCIM) methods were used. In these methods, the test strain was incubated with a carbapenem antibiotic and growth inhibition was assessed. EDTA solution (initial concentration 0.1 mol/L, 10 μL could be added dropwise, final concentration 292 μg/tablet) was added for modified carbapenem inactivation + EDTA (eCIM). The addition of EDTA in the eCIM helped to detect metallo-beta-lactamases. An increase in the zone of inhibition by≥5 mm in the eCIM compared to the mCIM was interpreted as a positive result for metallo-beta -lactamases.

However, there was currently no definitive phenotypic detection method specifically designed for class D carbapenemases. In the absence of a specific test, the elimination method could be used. If a strain was not inhibited by a class A or class B inhibitor, it could be presumed to possess a class D carbapenemase.

#### Antimicrobial susceptibility test

Bacterial resistance was detected using the VITEK2 system from BioMérieux of France. The VITEK2 determined the minimum inhibitory concentration (MIC) values of antibiotics. Additionally, the antibiotic paper diffusion method was employed to determine the zone of inhibition in millimeters (mm) for each antibiotic. The drug susceptibility paper used in the antibiotic paper diffusion method was supplied by OXOID, UK. The results of both methods were then confirmed according to CLSI 2020 guidelines (CLSI, 2022). A total of twenty-three antibiotics and antibiotics combined with enzyme inhibitors were tested, as listed in **Table 1**. *E. coli* ATCC 25922 was used for the quality control.

**Table 1:** MICs and genetic profiles of F11 *K. pneumoniae*.

Antimicrobial agents	MIC(mg/L)	Mechanism of resistance/location of resistance gene
**Aminoglycoside**		
Amikacin	≥6	*aadA2*, *rmtB* **(Plasmid B)**
**β-lactams**		
Urtapenem	≥8	*bla*_OXA-1_, *bla*_CTX-M-14_ **(Plasmid A)**/*bla*_TEM-1_, *bla*_SHV-134_ **(Plasmid B)**/*bla*_SHV-66_, *bla*_NDM-1_ **(Plasmid C)**
Imipenem	≥2	*bla*_OXA-1_, *bla*_CTX-M-14_ **(Plasmid A)**/*bla*_TEM-1_, *bla*_SHV-134_ **(Plasmid B)**/*bla*_SHV-66_, *bla*_NDM-1_ **(Plasmid C)**
Ampicillin	≥64	Intrinsic resistance; *bla*_SHV-187_ **(Chromosome)**
Aztreonam	≥32	*bla*_OXA-1_, *bla*_CTX-M-14_ **(Plasmid A)**/*bla*_TEM-1_, *bla*_SHV-134_ **(Plasmid B)**/*bla*_SHV-66_, *bla*_NDM-1_ **(Plasmid C)**
Cefepime	≥32	*bla*_OXA-1_, *bla*_CTX-M-14_ **(Plasmid A)**/*bla*_TEM-1_, *bla*_SHV-134_ **(Plasmid B)**/*bla*_SHV-66_, *bla*_NDM-1_ **(Plasmid C)**
Cefotaxime	≥64	*bla*_OXA-1_, *bla*_CTX-M-14_ **(Plasmid A)**/*bla*_TEM-1_, *bla*_SHV-134_ **(Plasmid B)/***bla*_SHV-66_, *bla*_NDM-1_ **(Plasmid C)**
Ceftazidime	≥64	*bla*_OXA-1_, *bla*_CTX-M-14_ **(Plasmid A)**/*bla*_TEM-1_, *bla*_SHV-134_ **(Plasmid B)**/*bla*_SHV-66_, *bla*_NDM-1_ **(Plasmid C)**
Ceftriaxone	≥64	*bla*_OXA-1_, *bla*_CTX-M-14_ **(Plasmid A)**/*bla*_TEM-1_, *bla*_SHV-134_ **(Plasmid B)**/*bla*_SHV-66_, *bla*_NDM-1_ **(Plasmid C)**
Cefuroxime	≥64	*bla*_OXA-1_, *bla*_CTX-M-14_ **(Plasmid A)**/*bla*_TEM-1_, *bla*_SHV-134_ **(Plasmid B)**/*bla*_SHV-66_, *bla*_NDM-1_ **(Plasmid C)**
Amoxicillin/Clavulanic acid	≥32	*bla*_OXA-1_, *bla*_CTX-M-14_ **(Plasmid A)**/*bla*_TEM-1_, *bla*_SHV-134_ **(Plasmid B)**/*bla*_SHV-66_, *bla*_NDM-1_ **(Plasmid C)**
Cefoperazone/Sulbactam	≥64	*bla*_OXA-1_, *bla*_CTX-M-14_ **(Plasmid A)**/*bla*_TEM-1_, *bla*_SHV-134_ **(Plasmid B)**/*bla*_SHV-66_, *bla*_NDM-1_ **(Plasmid C)**
Cefoperazone/Avibactam (30/20 μg)	6*	blaO_XA-1_, *bla*_CTX-M-14_ **(Plasmid A)**/*bla*_TEM-1_, *bla*_SHV-134_ **(Plasmid B)**/*bla*_SHV-66_, *bla*_NDM-1_ **(Plasmid C)**
**Cephamycin**		
Cefoxitin	≥64	*bla*_OXA-1_, *bla*_CTX-M-14_ **(Plasmid A)**/*bla*_TEM-1_, *bla*_SHV-134_ **(Plasmid B)**/*bla*_SHV-66_, *bla*_NDM-1_ **(Plasmid C)**
**Fluoroqinolones**		
Levofloxacin	≥8	qnrA1 **(Plasmid B)**, oqxB/oqxA **(Chromosome)**
Ciprofloxacin	≥4	qnrA1 **(Plasmid B)**, oqxB/oqxA **(Chromosome)**
**Tetracycline**		
Tetracycline	≥64	tetA, tetD **(Plasmid B)**
**Glycylcycline**		
Tigecycline	≥8	tetA-tetR, tetD-tetR **(Plasmid B)**/oqxAB **(Chromosome)**
**Glycopeptide**		
Bleomycin	–	ble_MBL_ **(Plasmid B )**/ble_MBL_ **(PlasmidC)**
**Phenicol**		
Chloramphenicol	≥64	catB3 **(plasmid A)**/cmlA9 **(Plasmid B)**
**Sulfonamide**		
Trimethoprim-sulfamethoxazole	≥320	sul1 (Plasmid A)/sul1, dfrA12 (plasmid B)
**Macrolide**		
Erythromycin (15 μg)	20*	
**Lincomycin**		
Lincomycin (15 μg)	6^*^	fosE **(Plasmid A)**/fosA **(Chromosome)**
**Disinfecting agent and antiseptic**		
–	–	qacEdelta1 **(Plasmid A, Plasmid B)**

*Disc diffusion method.Bold values/numbers were used to highlight which plasmid these resistance genes exist on.

#### Sequencing and sequence assembly

The genomic sequencing of strain F11 was performed on the third-generation PacBio Sequel II platform (Pacific Biosciences, CA, USA) and the second-generation sequencing Illumina NovaSeq 6000 platform (Illumina, San Diego, USA) using a DNA library with an average size of ~15 kbp (range 10 to 20 kbp), and on a ~400 bp fragment library (range 150 bp to 600 bp). To ensure data quality, the raw data obtained from the PacBio Sequel II platform was trimmed using Canu v2.2 (https://github.com/marbl/canu) to obtain high-quality clean reads. On the other hand, the paired-end short Illumina reads were subjected to “*de novo*” assembly using either Unicycler v0.4.5 (https://github.com/rrwick/Unicycler) or SPAdes v3.15.3 (https://github.com/ablab/spades). After the assembly, sequence corrections were performed at the post-assembly level using Pilon v1.24 (https://github.com/broadinstitute/pilon) using the second generation sequencing reads. Overall, these multistep methods were able to generate reliable and accurate DNA sequences for strain F11.

#### Sequence annotation and comparison in detail

Open reading frames (orfs) and pseudogenes were predicted using *RAST2.0* (Liang et al., 2015), *BLASTP/BLASTN* (Boratyn et al., 2013), *UniProtKB/Swiss-Prot* (Boutet et al., 2016), and *RefSeq* databases (O’Leary et al., 2016). Resistance genes, mobile elements, and other features were annotated using online databases such as *CARD 2023* (Alcock et al., 2023), *ResFinder4.0* (Bortolaia et al., 2020), IS*finder* (Siguier et al., 2006), *INTEGRALL* (Moura et al., 2009), and the *Tn* Number Registry (Tansirichaiya et al., 2019). The virulence genes were predicted using *VFDB 2022* (Liu et al., 2022) (http://www.mgc.ac.cn/VFs/main.htm) and *BacWGSTdb 2.0* (Feng et al., 2021) (http://bacdb.cn/BacWGSTdb/index.php). Clonal MLST was confirmed using *MLST2.0* (https://cge.food.dtu.dk/services/MLST/) and *BacWGSTdb 2.0* (Feng et al., 2021). The type of incompatible (Inc) plasmid was determined based on the comparative analysis of replication genes using *BacWGSTdb 2.0* (Feng et al., 2021). *MUSCLE 3.8.31* (Edgar, 2004) and BLASTN were used for multiple and pairwise sequence comparisons such as plasmid pA_F11, plasmid pB_F11, and plasmid pC_F11 with their closely related plasmids. Circos plots of plasmids were drawn with CGView (Stothard et al., 2019). Partial sequences of plasmids were compared using BLASTN to calculate identity and coverage, followed by heatmap with the R package pheatmap (https://CRAN.R-project.org/package=pheatmap). All comparative figures were generated using the R package genoPlotR v0.8.11 (http://genoplotr.r-forge.r-project.org/) and edited using Inkscape v0.48.1 (https://inkscape.org/en). These tools and databases were used to analyze and visualize various genomic features.

#### Nucleotide sequence accession numbers

The sequences of F11_Chromosome, plasmid pA_F11, plasmid pB_F11, plasmid pC_F11 and plasmid pD_F11 were deposited on the GenBank database as the accession numbers of CP092901.1, CP092902.1, CP092903.1, CP092904.1 and CP092905.1, respectively. The GenBank accession numbers of the related plasmids compared with plasmid pA_F11, plasmid pB_F11 and plasmid pC_F11 were also listed in **Table 2**.

**Table 2:** Profiles of the plasmids studied in the paper.

No.	Plasmid	strain	Isolate source	Type	Size (kb)	GC%	status	Accession no.
1	Plasmid pA_F11	*Klebsiella pneumoniae*	Patient’s sputum (Taizhou, China)	IncFIB/ IncHI1B	221.757	46.15	Complete	CP092902.1
2	pC105-NDM1- IncHI3	*Klebsiella pneumoniae*	Patient’s urine (India)	IncFIB/ IncHI1B	300.876	46.62	Complete	MN240794.1
3	pKmfe267-1	*Klebsiella pneumoniae*	Patient’s urine (Hengyang, China)	IncFIB/ IncHI1B	251.953	47.33	Complete	CP071394.1
4	pNH34.1	*Klebsiella pneumoniae*	Patient’s sputum (Chiang Mai, Thailand)	IncFIB/ IncHI1B	300.327	46.56	Complete	CP034406.1
5	pCMC432M_P1	*Klebsiella pneumoniae*	Patient’s endotracheal aspirate (Christian medical college, India)	IncFIB/ IncHI1B	285.823	46.33	Complete	CP079156.1
6	Plasmid pB_F11	*Klebsiella pneumoniae*	Patient’s sputum (Taizhou, China)	IncFIB/ IncHI1B	159.322	54.33	Complete	CP092903.1
7	p362713-FIIK	*Klebsiella pneumoniae*	Patient (Zhejiang, China)	IncFIB/ IncFII	183.189	53.35	Complete	MN823999.1
8	p1_020049	*Klebsiella pneumoniae*	Patient’s pus (Leshan, China)	IncFIB/ IncFII	182.097	52.15	Complete	CP028784.1
9	pKp_Goe_629-1	*Klebsiella pneumoniae*	Patient’s urine (Goettingen, Germany)	IncFIB/ IncFII	260.772	52.67	Complete	CP018365.1
10	p1_020135	*Klebsiella pneumoniae*	Patient (Sichuan, China)	IncFIB/ IncFII	179.816	52.22	Complete	CP037966.1
11	Plasmid pC_F11	*Klebsiella pneumoniae*	Patient’s sputum (Taizhou, China)	IncX3	92.904	49.83	Complete	CP092904.1
12	pKP04NDM	*Klebsiella pneumoniae*	Patient’s urine (China)	IncX3	50.034	49.04	Complete	KU314941.1
13	pC46-NDM11	*Citrobacter freundii*	Patient’s urine (China)	IncX3	50.035	49.04	Complete	MW269623.1
14	p309074-NDM	*Klebsiella pneumoniae*	Patient (Heibei, China)	IncX3	50.035	49.04	Complete	MH909346.1
15	pP10159-1	*Citrobacter freundii*	Patient (Chongqing, China)	IncX3	50.034	49.04	Complete	MF072961.1

## Results

### Results of antimicrobial susceptibility test, enzymatic property, and transferrable feature

The 16S rRNA genome sequence of strain F11 was confirmed as *K. pneumoniae* by BLAST and average nucleotide homology analysis. The results of drug susceptibility testing for strain F11 were shown in **Table 1**. The strain was highly resistant to all conventional antibiotics except erythromycin (20 mm, susceptible), especially cefoperazone/avibactam (6 mm, resistant) and tigecycline (≥8, resistant) (**Table 1**).

Enzyme analyses revealed that strain F11 produced class A beta-lactamase (positive ESBls) and class B metallo-beta-lactamase (class B carbapenemase); whereas class D carbapenemase was identified by analysis of plasmid sequence alignment.

In bacterial conjugative transfer and electroporation assays, transconjugants carrying the plasmid pA_F11 and plasmid pB_F11 were successfully obtained with average conjugation frequencies of 2.91×10^-4^ and 1.56×10^-4^, respectively. However, both the plasmid pC_F11 and plasmid pD_F11 could not be transferred by conjugation or electroporation.

### Overview of structural features for F11_chromosome

The F11_Chromosome with a length of 5.286326 Mbp, carried four antibiotic resistance genes, including *oqxB/A*, *bla*_SHV-187_, and *fosA* (**Supplementary Table S1**). as well as a series of virulence genes (**Supplementary Table S2**). Several categories of virulence gene, such as adhesion, biofilm, regulatory, immune modulation, type VI secretion system (T6SS), siderophore uptake system, and antimicrobial activity, exhibited more than 96.5% sequence identity (**Supplementary Figure S2** and **Supplementary Table S2**), suggesting a high degree of conservation and similarity among these gene categories in the F11_Chromosome. However, the category of virulence genes for the common pilus of *E. coli* showed lower sequence identities, ranging from 86.5% to 91.0% (**Supplementary Figure S2** and **Supplementary Table S2**), indicating some level of divergence or variation in these gene category within the F11_Chromosome.

### Characterization of plasmid pA_F11 and comparison of plasmid pA_F11 with related plasmids: pC105-NDM1-IncHI3, pNH34.1, pKmfe267-1, and pCMC432M_P1

**Figure 1:**
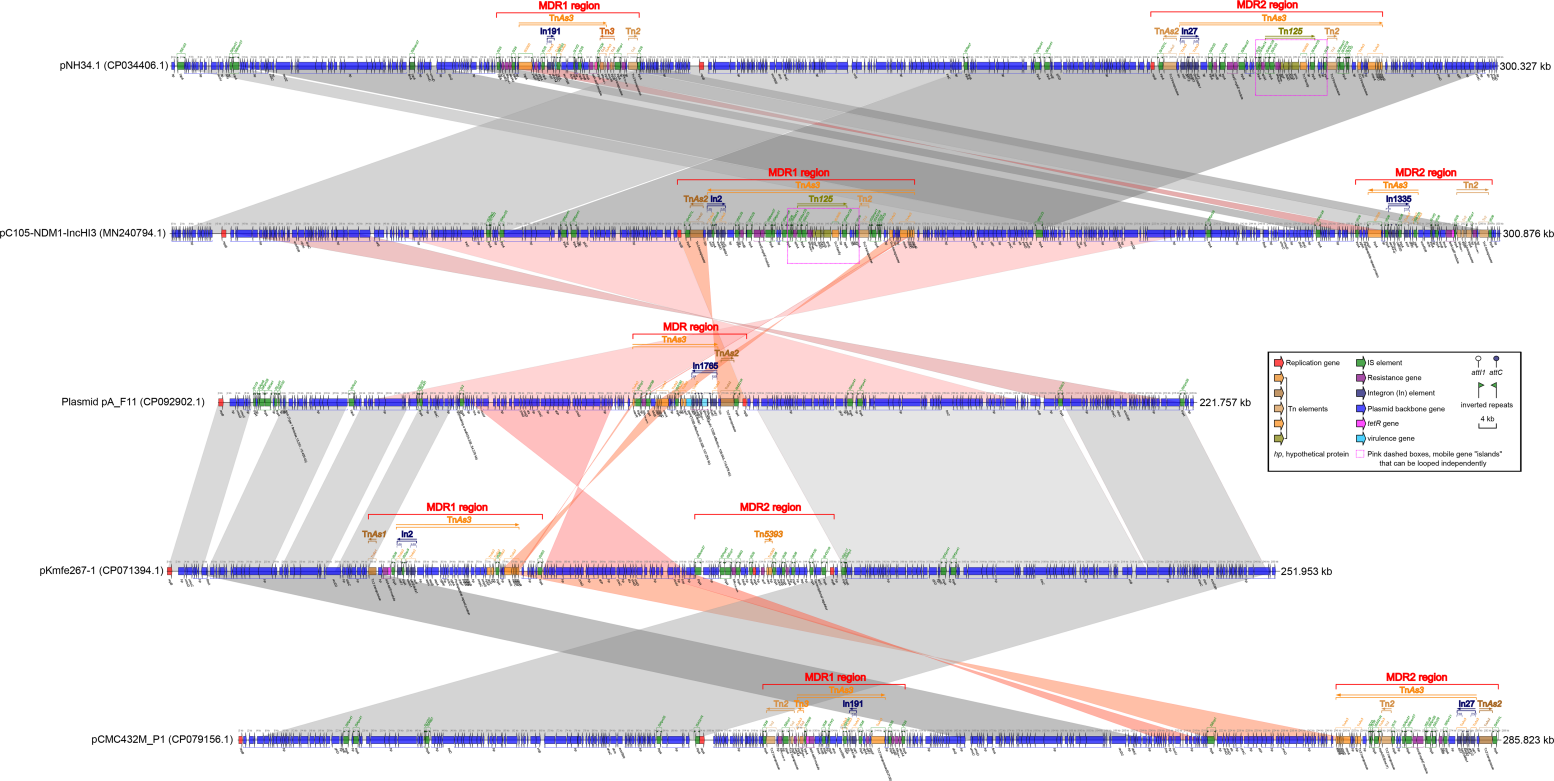
Comparison of plasmid pA_F11 with related plasmids. The shadow represents > 95% identity, while light gray represents the positive direction, and light pink refers to the opposite direction. The figures were created by the R pacakge genoPlotR v0.8.11 software (http://genoplotr.r-forge.r-project.org/).

Plasmid pA_F11, classified as IncFIB/IncHI1B type (**Table 2**), had a length of 221.757 kbp. It contained a MDR region that spans approximately 25.8 kbp. Additionally, the plasmid carried two *repB* genes, four virulence genes (*fimA*, *tssM*, *ipaH*, and *ipaH*), a number of ISs, and plasmid backbone genes (**Figure 1**).

The *fimA* gene, which belonged to the type 1 fimbriae, was located between the 15.251 kbp and 15.409 kbp sites. The *tssM* gene was covered by a hypothetical protein and was located between the 54.328 kbp and 54.378 kbp sites. Two copies of the *ipaH* gene were found within the MDR region, occupying positions from 105.926 kbp to 107.254 kbp and from 109.645 kbp to 110.978 kbp. These *ipaH* genes functioned as type III secretion system (T3SS) effectors (**Figure 1**). The MDR region of plasmid pA_F11 consisted of the transposons Tn*As3*, Tn*As2* and the rare integron In1765 (**Supplementary Table S3**). Within this region, several antibiotic resistance genes were present, including *bla*_CTX-M-14_, *sul1*, a *catB3* gene cassette, a *bla*_OXA-1_ gene cassette, and a *fosE* gene cassette. Notably, the *bla*_OXA-1_ gene cassette was split into two parts by the *gcu206* gene cassette (*ipaH*) in the region of 109.645-110.978 kbp, which was a rare occurrence (**Figure 1** and **Supplementary Table S3**).

Comparative analysis of plasmid pA_F11 with pC105-NDM1-IncHI3 revealed minimal similarity between the MDR region of plasmid pA_F11 and the MDR1 region of pC105-NDM1-IncHI3. There was no similarity between the MDR region of plasmid pA_F11 and the MDR2 region of pC105-NDM1-IncHI3. However, the plasmid backbone region surrounding the MDR region of pA_F11 showed perfect identity with that surrounding the MDR1 region of pC105-NDM1-IncHI3 (**Figure 1**).

When comparing plasmid pA_F11 with pKmfe267-1, it was observed that the MDR region of plasmid pA_F11 exhibited minimal similarity to the MDR1 and MDR2 regions of pKmfe267-1. On the other hand, the backbone region of plasmid pA_F11 showed high identity with that of pKmfe267-1 (**Figure 1**). Comparing pC105- NDM1-IncHI3 with pNH34.1, the two plasmids exhibited a high degree of sequence identity and coverage. However, certain sequences in the front and back positions showed alterations, including shifts in two MDR regions of the plasmids. Interestingly, both the MDR1 region of pC105-NDM1-IncHI3 and the MDR2 region of pNH34.1 carried identical *bla*_NDM-1_ and Tn*125* components (**Figure 1**). In the comparison of pKmfe267-1 with pCMC432M_P1, the backbone regions of both plasmids displayed excellent identity and coverage. However, the two MDR regions of pKmfe267-1 were not similar to those of pCMC432M_P1. Additionally, there were sequence inversions and shifts in the front and back of the backbone regions of the two plasmids (**Figure 1**).

### Characterization of plasmid pB_F11 and comparison of plasmid pB_F11 with Related Plasmids: p362713-FIIK, p1_020049, pKp_Goe_629-1, and p1_020135

**Figure 2:**
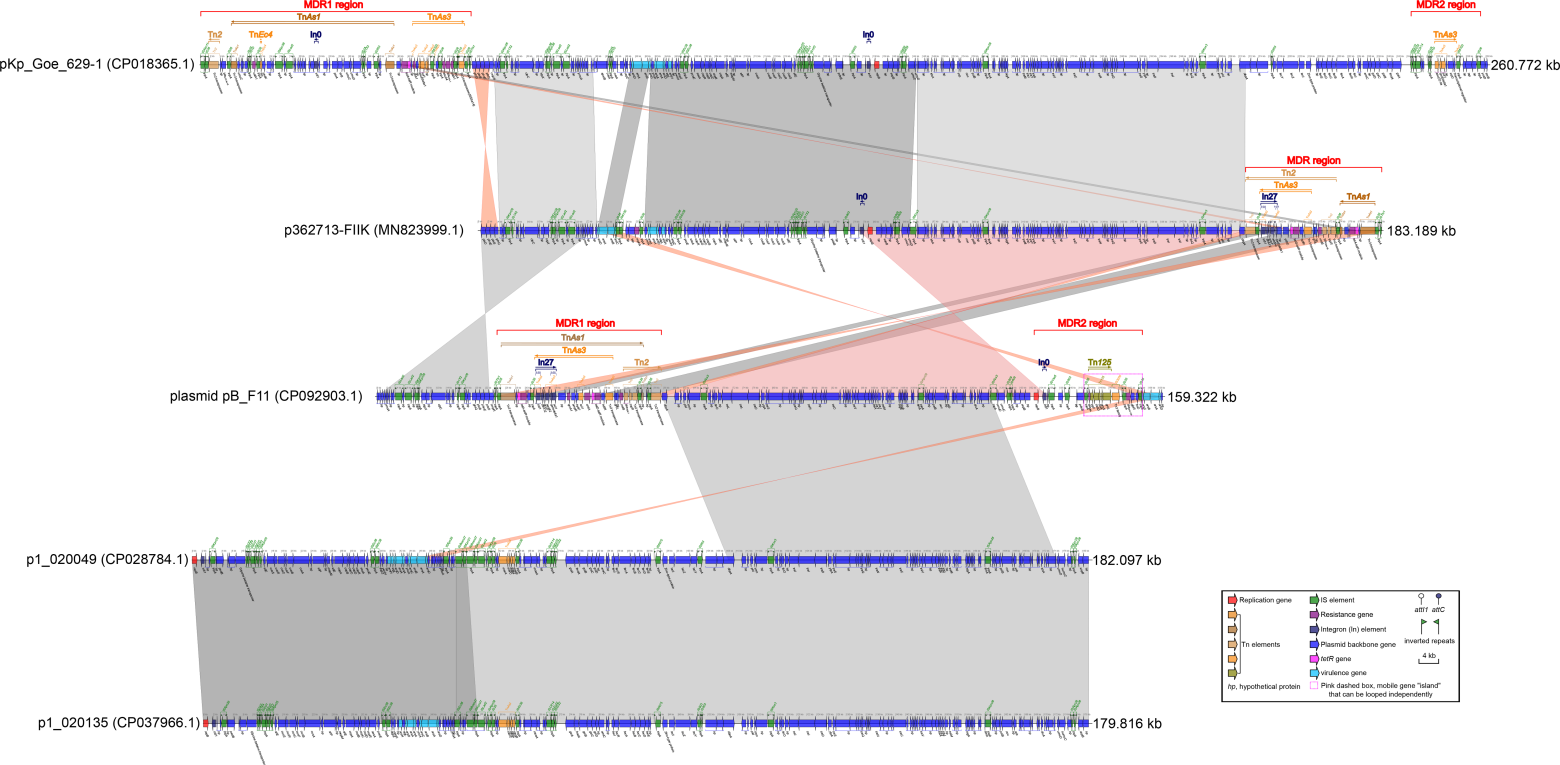
Comparison of plasmid pB_F11 with related plasmids. The shadow represents > 95% identity, while light gray represents the positive direction, and light pink refers to the opposite direction. The figures were also created by the R pacakge genoPlotR v0.8.11 software (http://genoplotr.r-forge.r-project.org/).

**Figure 3:**
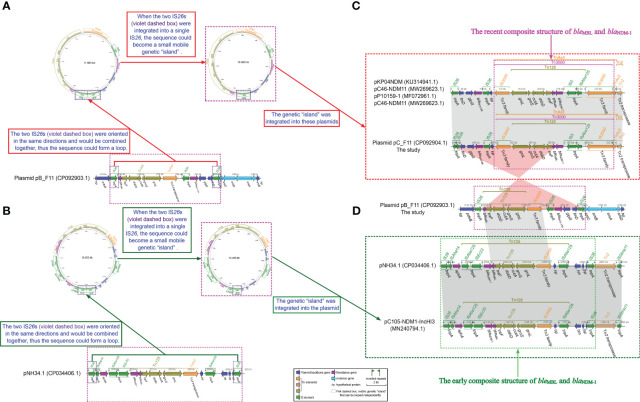
Schematic diagram of whether small loop intermediates could be formed by the integration of IS*26*s. **(A)** Schematic representation of a small loop intermediate that could be formed for plasmid pB_F11. **(B)** Schematic representation of a small loop intermediate that could be formed for pNH34.1. **(C)** comparisons of plasmid pC_F11, pNH34.1 and related plasmids, respectively, and **(D)** comparisons of plasmid pB_F11 with plasmid pC_F11 and pNH34.1. The diagram was created by the R package genoPlotR v0.8.11 software (http://genoplotr.r-forge.r-project.org/). The small loop diagrams were established using CGview v2.0.3 (https://github.com/paulstothard/cgview).

Plasmid pB_F11, classified also as IncFIB/IncHI1B type (**Table 2**), had a length of 159.322 kbp. It contained two MDR regions, *a repB* gene, four virulence genes related to heavy metal resistance (*arsC*, *arsB*, *arsA*, *and arsD* virulence genes), several ISs, and plasmid backbone genes (**Figure 2**). The MDR1 region was composed of transposons Tn*As3*, Tn*As2*, Tn*2*, and In27 (**Supplementary Table S3**). This region carried several antibiotic resistance genes, including *tetA*, a *dfrA12* gene cassette, an *aadA2* gene cassette, *sul1*, *qnrA1*, *cmlA*, *tetD*, *rmtB*, and *bla*_TEM-1_ (**Figure 2**). The MDR2 region of plasmid pB_F11 consisted of transposons Tn*125*, Tn*As3*, and In0. Interestingly, this region did not harbor the *bla*_NDM-1_ gene, unlike other plasmids that typically carried it. However, all other Tn*125* components in pB_F11 were identical to those found in plasmids known to carry *bla*_NDM-1_ (**Figure 2**). It suggested that plasmid pB_F11 might have lost the *bla*_NDM-1_ gene during its evolutionary process (**Figure 3**).

Comparing plasmid pB_F11 with p362713-FIIK, it was observed that the MDR1 region of plasmid pB_F11 showed some similarity to the MDR region of p362713-FIIK. Additionally, the backbone region of plasmid pB_F11 surrounding the MDR1 region was also similar to that of p362713-FIIK, with most sequences reversed (**Figure 2**). When comparing plasmid pB_F11 with p1_020049, part of the backbone region of plasmid pB_F11 was identical to that of p1_020049, except for the MDR region (**Figure 2**). In the comparison of p362713-FIIK with pKp_Goe_629-1, the backbone region of p362713-FIIK, which contained only one MDR region, showed good identity with that of pKp_Goe_629-1, which contained two MDR regions. However, there was no similarity between their respective MDR regions (**Figure 2**). Comparing p1_020049 with p1_020135, a high degree of identity was observed between the two plasmids. However, neither of them contained an MDR region (**Figure 2**).

### Characterization of plasmid pC_F11 and comparison of plasmid pC_F11 with related plasmids: pKP04NDM, pC46-NDM11, p309074-NDM, and pP10159-1

**Figure 4:**
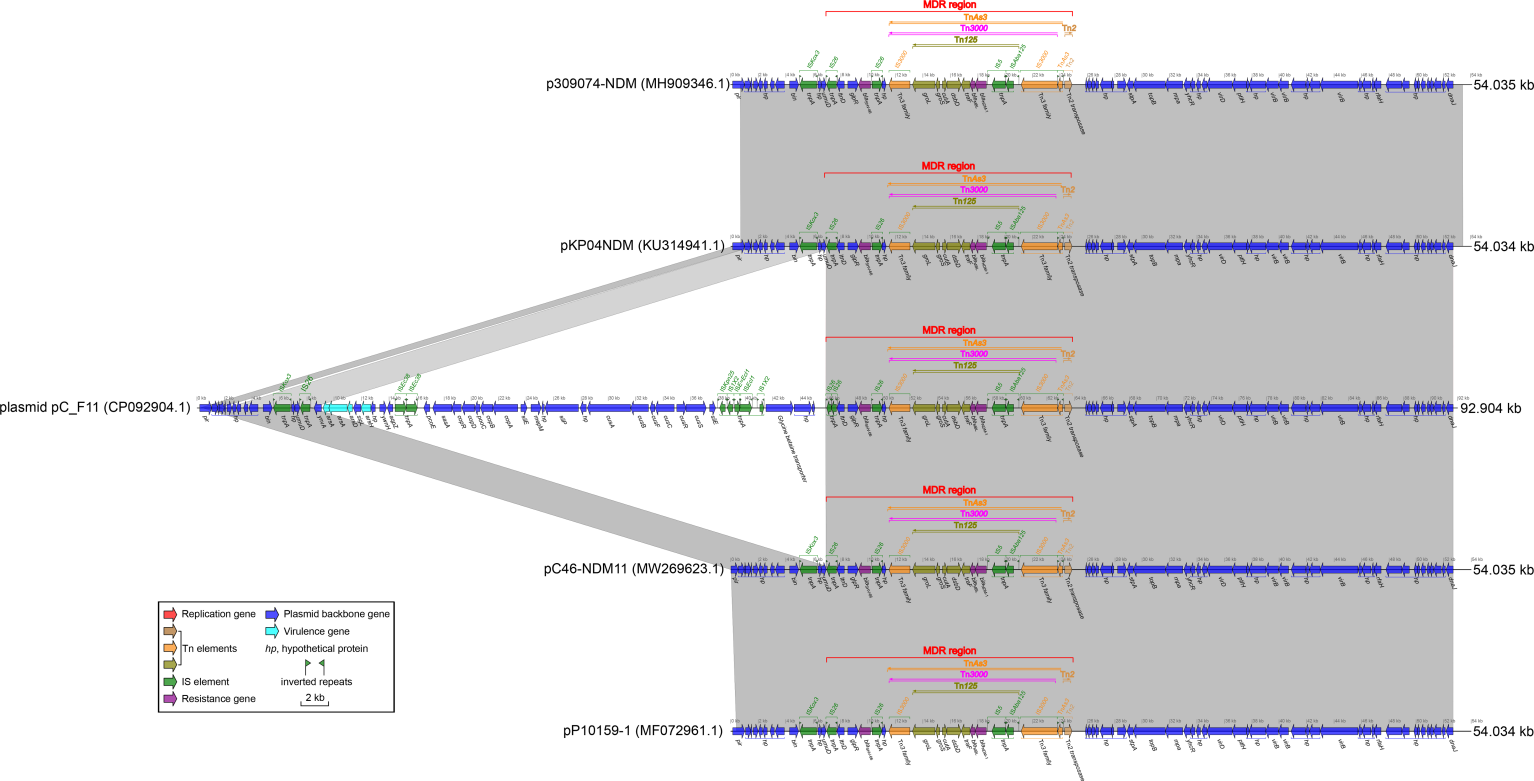
Comparison of plasmid pC_F11 with related plasmids. The shadow represents > 95% identity, while light gray represents the positive direction, and light pink refers to the opposite direction. The Figures were also created by the R pacakge genoPlotR v0.8.11 software (http://genoplotr.r-forge.r-project.org/).

Plasmid pC_F11, classified as an IncX3 type (**Table 2**), had a length of 92.904 kbp. It contained an MDR region, four virulence genes related to heavy metal resistance (*arsC*, *arsB*, *arsA*, *and arsD* virulence genes), several ISs, and plasmid backbone genes. However, no *repB* gene was found in this plasmid (**Figure 4**). The MDR region of plasmid pC_F11 consisted of transposons Tn*As3*, Tn*3000*, Tn*125*, and Tn*2*. This region involved antibiotic resistance genes such as *bla*_SHV-66_, *ble*_MBL_, and *bla*_NDM-1_ (**Figure 4**). Notably, the plasmid carried an identical *bla*_NDM-1_ gene and its associated Tn*125* components, which were part of Tn*3000* (**Figure 4**).

When comparing plasmid pC_F11 with plasmids pKP04NDM, pC46-NDM11, p309074-NDM, and pP10159-1, a high degree of identity was observed. However, there was an interesting difference in plasmid pC_F11, where an approximately 37.69 kbp-long sequence was inserted between the two IS*26* sites at positions 7.537 to 8.241 kbp and 45.931 to 46.260 kbp (**Figure 4**). Furthermore, the partial sequence of plasmid pB_F11 (indicated by pink dashed boxes in **Figure 3C**) was almost identical to the sequences of plasmids: plasmid pC_F11, p362713-FIIK, p1_020049, pKp_Goe_629-1, and p1_020135, all of which carried the *bla*_NDM-1_ gene. The only difference was that plasmid pB_F11 lacked the *bla*_NDM-1_ gene (**Figure 3C**).

### Characterization of plasmid pD_F11

Plasmid pD_F11 was characterized as a plasmid fragment with a length of 2.927 kbp. It consisted of only four hypothetical proteins and did not contain a *repA* gene or any antibiotic resistance genes (**Supplementary Figure S2**).

### Exploring the evolution of *bla*_NDM_ and its Tn*125* components

An detailed analysis of the plasmid pB_F11 sequence revealed that from 143.212 kbp to 154.876 kbp, it carried a genetic assay consisting of IS*26* (forward)–(*ble*_MBL_–*trpF*–*dsbB*–*cutA*–*groS*–*groL*) (Tn*125*, forward)–IS*3000*(forward)–IS*26* (reverse)–*bla*_SHV-134_ (forward)–*glpR* (reverse)–*ltnD* (forward)–IS*26* (forward), but did not contain the *bla*_NDM_ gene (**Figure 3A**). Upon integration of the two forward IS*26* elements into a single IS26, it formed independent genetic “islands” (**Figure 3A**). This identical structure was also found to be integrated in plasmids such as pKP04NDM, pC46-NDM11, pP10159-1, pC46-NDM11, and plasmid pC_F11 (**Figure 3C**). By performing a BLASTN analysis using the sequence of the genetic “islands”, it was discovered that this integratable structure was present in 50 plasmids. Interestingly, all 50 of these plasmids carried the *bla*_NDM_ gene (**Supplementary Figure S1**; **Supplementary Tables S4**).

In the case of the pNH34.1 sequence, from 245.506 kbp to 261.607 kbp, it contained a genetic assay as follows, IS*26* (forward)–IS*Aba14* (forward)–*aphA* (forward)–IS*Aba125* (forward)–IS*Ec33* (forward)–(*bla*_NDM-1_–*ble*_MBL_–*trpF*–*dsbB*–*cutA*–*groS*–*groL*) (Tn*125*, forward)–IS*3000* (forward)-*hp* (reverse)–IS*Aba125* (forward)–*hin* (reverse)–*hp* (reverse)–*hp* (forward)–*hp* (forward)–*hp* (forward)–IS*26* (forward)(**Figure 3B**). The integration of two forward IS*26* elements into a single IS*26*, as observed in the pNH34.1 sequence, also led to the formation of independent genetic islands” (**Figure 4B**). This structure also appeared in plasmids pC105-NDM1- IncHI3 (**Figure 3C**). Sequence BLASTN analysis revealed that only pNH34.1 and pC105-NDM1-IncHI3 had this specific structure. None of the other plasmids examined showed an equivalent assay.

## Discussion

The *bla*_NDM_ gene was commonly found on plasmids containing different backbone components (Acman et al., 2022). IncHI-like plasmids, which were typically larger than 200 kbp in size, had a wide distribution in the host population (Rozwandowicz et al., 2018) and played a crucial role in the dissemination of heavy metal resistance genes and antibiotic resistance genes (Cain and Hall, 2012). For example, IncHI5-like plasmids were often associated with different carbapenemase genes (Zhu et al., 2020), and two IncHI5-like plasmids were previously reported to carry both carbapenem resistance genes and tigecycline resistance modules (Qin et al., 2021). To date, at least 20 different types of incompatible plasmids had been associated with the *bla*_NDM_ gene. These plasmids included IncFIB, IncFII, IncA/C (IncC), IncX3, IncH, IncL/M, as well as untyped plasmids (Acman et al., 2022).

Prior to 1985, the *bla*_NDM_ gene was typically found within the Tn*125* transposon, containing the IS*26* insertion sequence. However, the global prevalence of IS*26* did not occur until after 2015 (Acman et al., 2022). Tn*125* played a important role in the early plasmid-mediated dissemination of the *bla*_NDM_ gene (**Figure 3D**), but in recent years, it had been replaced by mobile elements such as Tn*3000* (Acman et al., 2022). The plasmids pC105- NDM-IncHI3 and pNH34.1, which showed high similarity to the non-MDR region of plasmid pA_F11, shared an identical relatively complete Tn*125* structure in the MDR1 region of pC105-NDM1-IncHI3 and the MDR2 region of pNH34.1. This structure represented an early composite structure of *ble*_MBL_ and *bla*_NDM-1_ between the two ends of IS*Aba15* (**Figure 3D**). Plasmid pC_F11 exhibited a high degree of identity in both the MDR and non-MDR regions with pKP04NDM, pC46-NDM11, p309074-NDM, and pP10159-1, except for the insertion of a non-MDR sequence of approximately 37.69 kbp in length. In the MDR region of these plasmids, the composite structure of *ble*_MBL_ and *bla*_NDM-1_, located between the two IS*3000* elements, contained a portion of the Tn*125* structure. This composite structure had been classified as the recently prevalent Tn*3000* structure (Acman et al., 2022) (**Figure 3C**). Several mobile elements were recognized to play a critical role in the dissemination of these genetic elements, including IS*Aba125*, IS*3000*, IS*26*, IS*5*, IS*CR1*, Tn*3*, Tn*125*, and Tn*3000* (Acman et al., 2022). Tn*3000* was flanked by two copies of IS*3000* and had a length of approximately 11.800 kbp (Campos et al., 2015). The first copy of IS*3000* truncated the upstream portion of the IS*Aba125* element, followed by the *bla*_NDM-1_ gene. Downstream of the *bla*_NDM-1_ gene, there was the *ble*_MBL_ gene, followed by the *trpF*, *dsbD*, and *cutA* genes. The *groL* and *groS* genes were linked to the second copy of IS*3000*, forming the complete Tn*3000* structure. This structure was also observed in plasmids such as pC_F11, pKP04NDM, pC46-NDM11, p309074-NDM, and pP10159-1 (**Figures 3C**, **4**).

Plasmid pA_F11 showed high sequence identity with related plasmids in the non-MDR regions, but low sequence identity in the MDR region. It was constructed mainly from a composite of Tn*As3*, Tn*As2*, and In1765. The sequence structure of plasmid pA_F11 with only one MDR region, especially In1765, was rare and had not been identified in the literature or GenBank. Plasmid pB_F11 consisted of two MDR regions, MDR1 and MDR2. The MDR regions of plasmid pB_F11 and plasmid p362713-FIIK shared identical In27 and part of the Tn*As3* components. The analysis revealed that the MDR2 region of plasmid pB_F11 independently formed a circular genetic “island” characterized as a mobile element. This genetic “island” contained genetic arrays including IS26–(*ble*_MBL_–*trpF*–*dsbB*–*cutA*–*groS*–groL)–IS*3000*–IS*26*–*bla*_SHV-134_–*glpR*–*ltnD*–IS*26* (**Figure 3**), which was part of the composite structure of *ble*_MBL_ and *bla*_NDM-1_, except for the absence of *bla*_NDM-1_. Further BLASTN analysis using these sequences of genetic “island” as templates, it showed highly consistent results, except that all 50 identified sequences carried the *bla*_NDM-1_ gene (**Supplementary Figure S1**; **Supplementary Table S3**). These sequences represented the popular composite structure observed in recent years (Acman et al., 2022). The results indicated that the MDR2 region of plasmid pB_F11 evolved as a unique structure due to the loss of the *bla*_NDM-1_ gene during the plasmid evolution process. Similarly, the MDR2 region of pNH34.1 and the MDR1 region of pC105-NDM1-IncHI3 independently developed circular genetic “islands” characterized as mobile elements (**Figure 3**). These genetic “islands” contained genetic arrays including IS*26*–IS*Aba14*–*aphA*–IS*Aba125*–IS*Ec33*–(*bla*_NDM-1_–*ble*_MBL_–*trpF*–*dsbB*–*cutA*–*groS*–*groL*)–IS*3000*–*hp*–IS*Aba125*–*hin*–*hp*–*hp*–*hp*–*hp*–IS*26*, which belonged to the early composite structure of *ble*_MBL_ and *bla*_NDM-1_ (Acman et al., 2022) (**Figure 3D**). BLASTN comparison using the sequences of genetic “islands” as templates revealed that only pNH34.1 and pC105-NDM1-IncHI3 had the identical structure, both representing the early composite structure of *ble*_MBL_ and *bla*_NDM-1_ (Acman et al., 2022) (**Figure 3D**). The MDR2 region of plasmid pB_F11 served as an independent mobile element while acting as a “bridge” connecting the early composite structure of *ble*_MBL_ and *bla*_NDM-1_ (**Figure 3D**) to the recent composite structure of *ble*_MBL_ and *bla*_NDM-1_ (Acman et al., 2022) (**Figure 3C**).

The strain F11 demonstrated a high level of antibiotic resistance, including resistance to conventional antibiotics such as cefoperazone/avibactam and tigecycline (**Table 1**). This resistance was attributed to the complex combination of transposons and gene cassettes carrying different antibiotic resistance and virulence genes on the F11_chromosome and several F11_plasmids. The strain was resistant to almost all antibiotics tested, limiting treatment options. Although erythromycin was found to be sensitive to strain F11 *in vitro*, clinical treatment with this antibiotic was ineffective. This might be due to the involvement of multiple virulence genes in the strain, which could contribute to its pathogenicity and ability to evade antibiotic treatment. As a result, no effective drugs were available to effectively treat infections caused by strain F11.

## Conclusions

The combination of complex overlapping transposons and gene cassettes, along with the synergistic effects of multiple antibiotic resistance and heavy metal resistance genes (virulence genes), contributed to the lack of an effective drug to treat infections caused by strain F11. It was essential to prevent the emergence and dissemination of strain F11 through strict control measures and the promotion of responsible antibiotic use.

## Data availability statement

The datasets presented in this study can be found in online repositories. The names of the repository/repositories and accession number(s) can be found below: The sequences of F11_Chromosome, plasmid pA_F11, plasmid pB_F11, plasmid pC_F11 and plasmid pD_F11 were deposited on the GenBank database as the accession numbers of CP092901.1, CP092902.1, CP092903.1, CP092904.1 and CP092905.1, respectively.

## Ethics statement

This study was approved by the Ethics Committee of Taizhou University, Zhejiang, China, and written informed consent was obtained from each of the participants in accordance with the Declaration of Helsinki. The rights of the research subjects were protected throughout, and we confirm that this study was conducted in our school. The use of human specimens and all related experimental protocols were approved by the Committee on Human Research of Taizhou University, and the protocols were carried out in accordance with approved guidelines.

## Author contributions

LM: Data curation, Formal analysis, Investigation, Methodology, Project administration, Software, Supervision, Visualization, Writing – review & editing, Funding acquisition. YQ: Data curation, Formal analysis, Investigation, Methodology, Resources, Software, Supervision, Validation, Visualization, Writing – review & editing. WW: Data curation, Formal analysis, Investigation, Methodology, Resources, Software, Supervision, Validation, Visualization, Writing – review & editing. DW: Conceptualization, Data curation, Formal analysis, Funding acquisition, Investigation, Methodology, Project administration, Resources, Software, Supervision, Validation, Visualization, Writing – original draft, Writing – review & editing.

## Supplementary material

Supplementary Figure 1 Heatmap analysis of sequence coverage and identity using BLASTN with sequence of genetic “island” from plasmid pB_F11 as a template. The figure was created with the R package pheatmap (https://CRAN.R-project.org/package=pheatmap) base on **Supplementary Table S4**.

Supplementary Figure 2 Distribution of the location for virulence genes and antibiotic resistance genes on F11_chromosome.

Supplementary Figure 3 Circle diagram of plasmid pD_F11.
