# Supplementary material for: Characterization of Klebsiella pneumoniae carrying the blaNDM-1 gene in IncX3 plasmids and the rare In1765 in an IncFIB-IncHI1B plasmid
Source: Front Cell Infect Microbiol. 2024 Jan 11;13:1324846. doi: 10.3389/fcimb.2023.1324846 (PMC10808583; doi:10.3389/fcimb.2023.1324846)
Supplement: Supplementary file 1 [file Table_1.docx]

| **Table S1 Profiles of antimicrobial resistance gene in F11 chromosome of *Klebsiella pnenmoniae*** | | | |
| --- | --- | --- | --- |
| **Antimicrobial resistance gene** | **Identity (%)** | **Position (bp)** | **Antimicrobial resistance category** |
| *oqxB* | 98.45 | 1184025..1187177 | quinolone |
| *oqxA* | 99.15 | 1187201..1188376 | quinolone |
| *bla*_SHV-187_ | 99.88 | 2769227..2770093 | beta-lactam |
| *fosA* | 98.81 | 4537851..4538270 | fosfomycin |

| **Table 2 Profiles of virlence gene in F11 chromosome of *Klebsiella pnenmoniae*** | | | | | | | | | | | |
| --- | --- | --- | --- | --- | --- | --- | --- | --- | --- | --- | --- |
| **Virulence gene** | | | | **Identity (%)** | | | | | **Position (bp)** | | **Functional annotation** |
| **Adherence** | | |  | | | | | | | | |
|  | *fimK* | | | 98.87 | | | | | 861339..862580 | | transcriptional regulator |
|  | *fimH* | | | 99.12 | | | | | 862748..863656 | | type 1 fimbrial adhesin precursor |
|  | *fimG* | | | 99.4 | | | | | 863671..864171 | | type 1 fimbrial minor component |
|  | *fimF* | | | 99.44 | | | | | 864184..864714 | | type 1 fimbrial minor component |
|  | *fimD* | | | 99.7 | | | | | 864722..867367 | | outer membrane usher protein |
|  | *fimC* | | | 99.86 | | | | | 867416..868141 | | periplasmic chaperone |
|  | *fimI* | | | 99.37 | | | | | 868170..868805 | | type 1 pilus biosynthesis fimbrial protein |
|  | *fimA* | | | 99.64 | | | | | 868777..869325 | | type 1 major fimbrial subunit precursor |
|  | *fimE* | | | 99.01 | | | | | 869805..870413 | | tyrosine recombinase |
|  | *fimB* | | | 99.5 | | | | | 870879..871484 | | tyrosine recombinase |
| **Biofilm** | |  | | | | | | | | | |
|  | *mrkA* | | | 99.67 | | | | | 876150..876758 | | type 3 fimbrial major pilin subunit MrkA |
|  | *mrkB* | | | 99.72 | | | | | 876854..877555 | | fimbrial chaperone protein MrkB precursor |
|  | *mrkC* | | | 99.56 | | | | | 877567..880053 | | fimbrial biogenesis outer membrane usher protein MrkC precursor |
|  | *mrkD* | | | 99.7 | | | | | 880044..881039 | | fimbrial adhesin protein precursor MrkD |
|  | *mrkF* | | | 100 | | | | | 881053..881688 | | type 3 fimbrial minor pilin subunit MrkF |
|  | *mrkJ* | | | 99.72 | | | | | 881723..882439 | | phosphodiesterase |
|  | *mrkI* | | | 99.49 | | | | | 882583..883167 | | LuxR family regulatory protein |
|  | *mrkH* | | | 99.86 | | | | | 883173..883883 | | transcriptional activator |
| **Regulation** | | |  | | | | | | | | |
|  | *rcsB* | | | 100 | | | | | 1617043..1617693 | | transcriptional regulator RcsB |
|  | *rcsA* | | | 100 | | | | | 1920312..1920935 | | transcriptional activator for ctr capsule biosynthesis |
| **Immune modulation** | | | | |  | | | | | | |
|  | *gnd* | | | 97.07 | | | | | 1790127..1791491 | | 6-phosphogluconate dehydrogenase |
|  | *ugd* | | | 97.51 | | | | | 1798298..1799422 | | UDP-glucose 6-dehydrogenase |
|  | *galF* | | | 98.77 | | | | | 1768594..1769490 | | UTP-glucose-1-phosphate uridylyltransferase subunit GalF |
|  | *cpsACP* | | | 91.17 | | | | | 1769883..1770481 | | phosphatase PAP2 family protein |
|  | *wbbM* | | | 98.15 | | | | | 1805967..1807862 | | glycosyltransferase |
|  | *wbbN* | | | 97.65 | | | | | 1809029..1809922 | | glycosyltransferase |
|  | *glf* | | | 98.44 | | | | | 1807878..1809032 | | UDP-galactopyranose mutase |
|  | *wbbO* | | | 97.71 | | | | | 1809935..1811025 | | glycosyltransferase family 1 protein |
| **Type VI secretion system (T6SS)** | | | | | | | | | |  | |
|  | *sciN/tssJ* | | | 100 | | | | | 2968688..2969230 | | type VI secretion system lipoprotein TssJ |
|  | *tssG* | | | 99.08 | | | | | 2969208..2970293 | | type VI secretion system baseplate subunit TssG |
|  | *tssF* | | | 99.37 | | | | | 2970257..2972011 | | type VI secretion system baseplate subunit TssF |
|  | *impA/tssA* | | | 96.8 | | | | | 2973278..2973683 | | type VI secretion system protein TssA |
|  | *icmF/tssM* | | | 98.76 | | | | | 2973685..2976754 | | type VI secretion protein TssM |
|  | *clpV/tssH* | | | 99.1 | | | | | 2985267..2987921 | | type VI secretion system ATPase TssH |
|  | *hcp/tssD* | | | 100 | | | | | 2988186..2988677 | | type VI secretion system protein, Hcp family |
|  | *dotU/tssL* | | | 99.42 | | | | | 2990385..2991074 | | type VI secretion system protein, DotU/TssL family |
|  | *vasE/tssK* | | | 99.33 | | | | | 2991071..2992414 | | type VI secretion system baseplate subunit TssK |
|  | *vipB/tssC* | | | 98.9 | | | | | 2992424..2993968 | | type VI secretion system contractile sheath large subunit VipB |
|  | *vipA/tssB* | | | 99.19 | | | | | 2994011..2994502 | | type VI secretion system contractile sheath small subunit VipA |
| **Siderophore uptake system** | | | | | | | |  | | | |
|  | *iroE* | | | 99.04 | | | | | 2692381..2693316 | | siderophore esterase IroE |
|  | *fepB* | | | 99.58 | | | | | 3758936..3759895 | | iron-enterobactin transporter periplasmic binding protein |
|  | *fepD* | | | 99.7 | | | | | 3761629..3762636 | | iron-enterobactin transporter membrane protein |
|  | *fepG* | | | 98.69 | | | | | 3762633..3763625 | | iron-enterobactin transporter permease |
|  | *fepA* | | | 99.28 | | | | | 3770072..3772300 | | outer membrane receptor FepA |
|  | *entA* | | | 98.34 | | | | | 3754336..3755120 | | 2,3-dihydroxybenzoate-2,3-dehydrogenase |
|  | *entB* | | | 98.23 | | | | | 3755094..3755941 | | 2,3-dihydro-2,3-dihydroxybenzoate synthetase, isochroismatase |
|  | *entE* | | | 97.89 | | | | | 3755955..3757562 | | enterobactin synthase subunit E |
|  | *entD* | | | 99.21 | | | | | 3772367..3772996 | | enterochelin synthetase component D |
|  | *entC* | | | 99.41 | | | | | 3757572..3758759 | | isochorismate synthase |
|  | *entF* | | | 99.05 | | | | | 3764481..3768362 | | enterobactin synthase subunit F |
|  | *fes* | | | 98.92 | | | | | 3768604..3769812 | | enterobactin/ferric enterobactin esterase |
|  | *ybdA* | | | 99.36 | | | | | 3760275..3761516 | | enterobactin exporter EntS |
| **Antimicrobial activity** | | | | | | |  | | | | |
|  | *acrA* | | | 99.92 | | | | | 3961789..3962982 | | acriflavine resistance protein A |
|  | *acrB* | | | 99.59 | | | | | 3963005..3966151 | | acriflavine resistance protein B |
| ***E. coli*** common pilus | | | | | |  | | | | | |
|  | *yagW/ecpD* | | | 89.44 | | | | | 4116072..4117672 | | polymerized tip adhesin of ECP fibers |
|  | *yagX/ecpC* | | | 87.48 | | | | | 4117705..4120211 | | *E. coli* common pilus usher EcpC |
|  | *yagY/ecpB* | | | 88.89 | | | | | 4120256..4120876 | | *E. coli* common pilus chaperone EcpB |
|  | *yagZ/ecpA* | | | 90.07 | | | | | 4120987..4121570 | | *E. coli* common pilus structural subunit EcpA |
|  | *ykgK/ecpR* | | | 86.75 | | | | | 4121645..4122225 | | regulator protein EcpR |

| **Table S3 Sequence analysis for In1765 from plasmid_pA_F11 and In27 from plasmid_pB_F11** | | | | | |
| --- | --- | --- | --- | --- | --- |
| **Integron Number** | **In1765** | |  | **In27** | |
| Pc variant | PcW | "-10": 112,032-112,037 bp |  | PcW | "-10": 33,092-33,097 bp |
|  |  | "-35": 112,055-112,060 bp |  |  | "-35": 33,069-33,074 bp |
| P2 promoter | Absent | |  | Absent | |
| PintI1 | OK | "-10": 111,913-111,918 bp |  | OK | "-10": 33,211-33,216 bp |
|  |  | "-35": 111,890-111,895 bp |  |  | "-35": 33,234-33,239 bp |
| 19 bp ORF11 dupl. | Yes | |  | No | |
| *IntI1* | IntI1_P32_H39_ | 111,945-112,958 bp |  | IntI1_P32_H39_ | 32,171-33,184 bp |
| *attI1* | 111,784-111,864 bp | |  | 33,265-33,326 bp | |
| Array of gene  cassettes | *fosE*_(111,328-111,789)_-*attC*_(111,322-111,381)_-*gcu206*_(111,050-111,327)_-*attC*_(111,044-111,104)_  -*bla*_OXA-1Δ(110,979-111,049)_-*ipaH*_(109,645-110,978)_- *bla*_OXA-1Δ(108,708-109,649)_  -*attC*_(108,702-108,791)_-*catB3*_(107,993-108,707)_-*attC*_(107,987-108,046)_-3'CS | |  | *dfrA12*_(33,321-33,904)_-*attC*_(33,821- 33,910)-_*gcuF*_(33,905-34,224)_-  *attC*_(34,171-34,230)_-*aadA2*_(34,225-35,080)_ -*attC*_(35,027-35,086)_-3'CS | |
| 5'-CS | 111790-111830 bp | |  | 33,280-33,320 bp | |
| CDS of GC | fosE_(111,376-111,780)_ /*gcu206*_(None)_ /*bla*_OXA-1(108,811-109,641)_ /*catB3*_(108,041-108,673)_ | |  | *dfrA12*_(33,329-33,826)_ /*gcuF*_(33,938-34,228)_ /*aadA2*_(34,234-35,025)_ | |
| *attC* | *attC*_fosE(111,784-111,789; 111,328-111,381)_ /*attC*_gcu206(111,322-111,327; 111,050-111,104)_  /*attC_bla_*_OXA-1(111,044-111,049; 108,708-108,791)_ /*attC*_catB3(108,702-108,707; 107,993-108,046)_ | |  | *attC*_dfrA12(33,321-33,326; 33,821-33,904)_ /*attC*_gcuF(33,905-33,910; 34,171-34,224)_  /*attC*_aadA_ _(34,225-34,230; 35,027-35,080)_ | |
| 3'-CS | 107,499-107,992 bp | |  | 35,081-35,574 bp | |
| GenBank accession number: Plasmid_pA_F11 (CP092902.1), Plasmid_pB_F11 (CP092903.1). | | | | | |
