# Supplementary figures and images for: Characterization of Klebsiella pneumoniae carrying the blaNDM-1 gene in IncX3 plasmids and the rare In1765 in an IncFIB-IncHI1B plasmid
Source: Front Cell Infect Microbiol. 2024 Jan 11;13:1324846. doi: 10.3389/fcimb.2023.1324846 (PMC10808583; doi:10.3389/fcimb.2023.1324846)

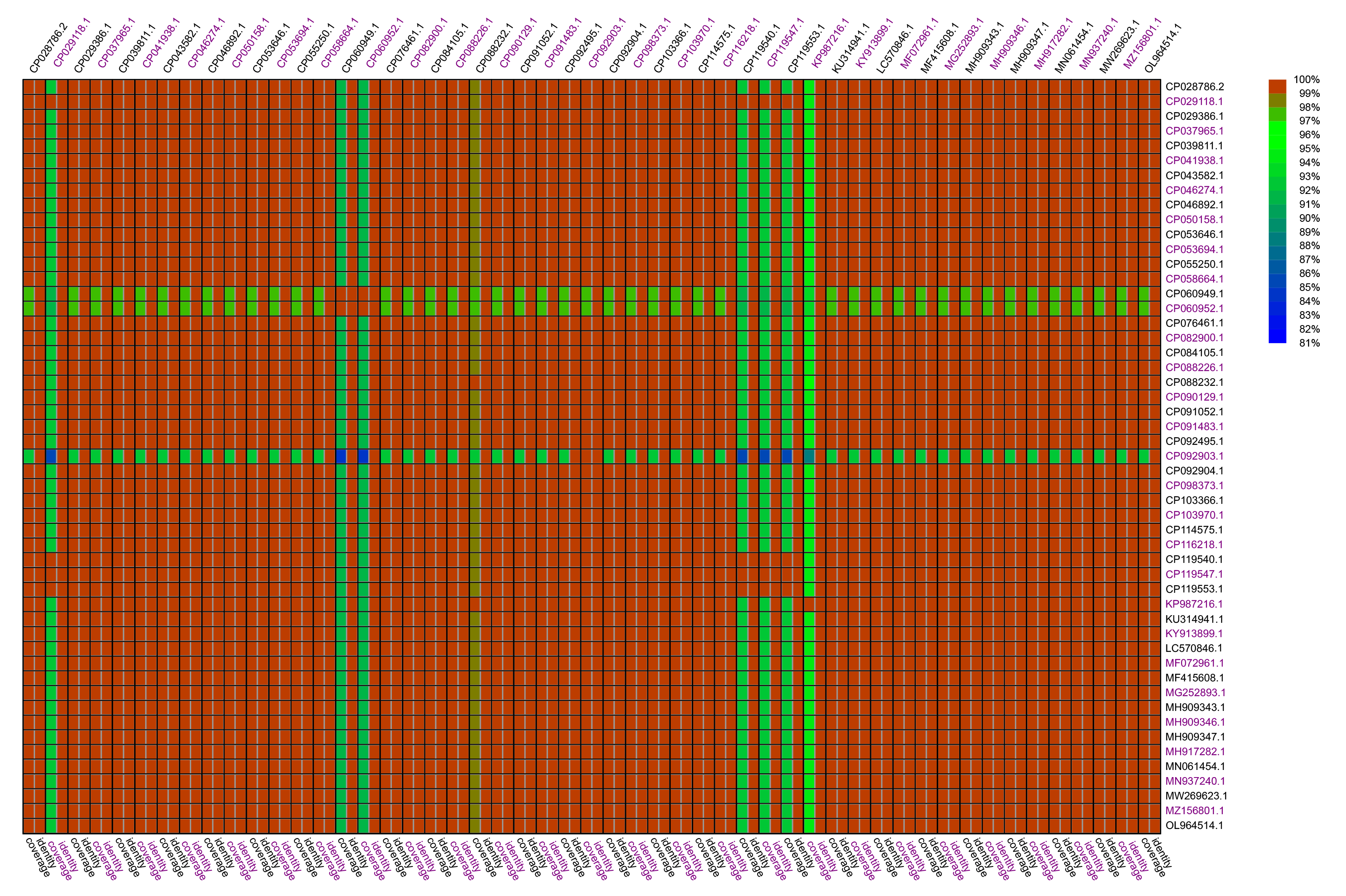

Supplement: Supplementary Figure 1 — Heatmap analysis of sequence coverage and identity using BLASTN with sequence of genetic “island” from plasmid pB_F11 as a template. The figure was created with the R package pheatmap (https://CRAN.R-project.org/package=pheatmap) base on Supplementary Table S4. [file Image_1.tif]

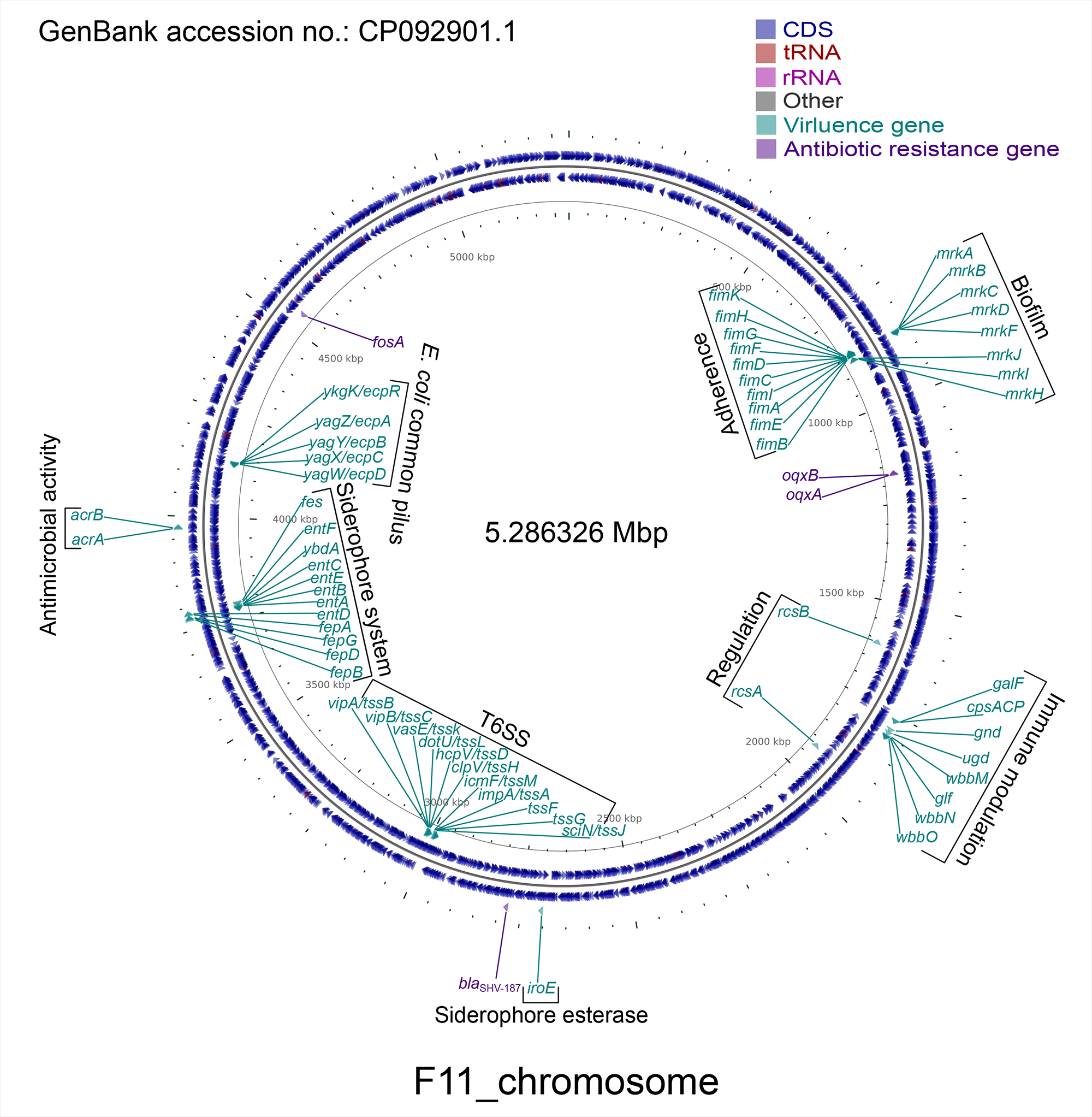

Supplement: Supplementary Figure 2 — Distribution of the location for virulence genes and antibiotic resistance genes on F11_chromosome. [file Image_2.tif]
